# Antifungal Effect of Brassica Tissues on the Mycotoxigenic Cereal Pathogen *Fusarium graminearum*

**DOI:** 10.3390/antibiotics11091249

**Published:** 2022-09-15

**Authors:** Samina Ashiq, Simon Edwards, Andrew Watson, Emma Blundell, Matthew Back

**Affiliations:** Agriculture and Environment Department, Harper Adams University, Newport, Shropshire TF10 8NB, UK

**Keywords:** biofumigant, sinigrin, head blight, *Brassica juncea*, *Eruca sativa*, *Raphanus sativus*

## Abstract

*Fusarium graminearum* is a globally important cereal pathogen, causing head blight in wheat, resulting in yield losses and mycotoxin contamination. Currently, triazole fungicides are used to suppress *Fusarium graminearum*, however, the declining effectiveness of triazoles and concerns over the safety of pesticides have led to the pursuit of safe alternative crop protection strategies such as biofumigation. In the present study, species belonging to Brassicaceae (*Brassica juncea*, *Raphanus sativus*, *Eruca sativa*) were assessed for their biofumigation potential against *F. graminearum* and the glucosinolate profile of the brassicas was determined. In Petri dishes, mycelial plugs of *Fusarium graminearum* were exposed to frozen/defrosted leaf discs of brassicas collected at early-leaf, stem-extension, and early-bud stages. Additionally, *F. graminearum* inoculum was incubated in soil amended with chopped tissues of brassicas in a closed jar experiment. Glucosinolate analysis of the leaf tissue of brassicas revealed that the total glucosinolate concentration of *B. juncea* ‘Brons’ increased with advancing growth stage (24.5–51.9 µmol g^−1^). *Brassica juncea* leaf discs were effective against mycelial growth, while the sinigrin content in the leaf tissue corresponded to the level of suppression. At the stem-extension and early-bud stages, *B. juncea* ‘Brons’ showed 87–90% suppression with four leaf discs, and 100% suppression with eight leaf discs. *Brassica juncea* ‘Caliente Rojo’ leaf discs collected at the stem-extension stage showed 94% inhibition with eight discs. In the closed jar experiment, each brassica species significantly suppressed *F. graminearum* inoculum by 41–55%. The findings suggest that the brassica species investigated in the present study could be effective in reducing the inoculum of *F. graminearum* in soil prior to cereal production.

## 1. Introduction

*Fusarium graminearum*, an ascomycete fungus, is the most prevalent and important pathogen of head blight in wheat and has been reported in all continents except Antarctica [1]. It is also an important causal agent of ear rot and stalk rot in maize [2]. Fusarium head blight can result in yield losses as high as 50% in cereals [3,4], although >70% yield losses were recorded in Argentina in 2012 [5]. In 2015/16, Fusarium head blight caused yield losses valued at $1.176 billion in the U.S. [6]. *Fusarium graminearum* not only causes yield losses but also economic and health losses due to mycotoxin production in cereals. The major mycotoxins produced by *F. graminearum* are deoxynivalenol and zearalenone. Deoxynivalenol induces vomiting, anorexia, reduces food intake, and causes hepatotoxic, immunotoxic, and neurovirulent effects [7]. Zearalenone, a mycoestrogen, has adverse effects on the reproductive system and is associated with the early onset of puberty in young children [8,9]. Contamination with concentrations exceeding the EU legal limits of 100 µg kg^−1^ zearalenone and 1250 µg kg^−1^ deoxynivalenol in wheat for human consumption were detected in 29% and 13% of wheat samples, respectively, in England in 2008 [10]. Moreover, 83% of durum wheat samples from Tunisia in 2007 [11] and 12.5% of maize samples from Serbia in 2011 [12] were found to be contaminated with deoxynivalenol at concentrations higher than the EU legal limit of 1750 µg kg^−1^ for these foodstuffs.

The use of fungicides, particularly triazoles, has been the main method of *F. graminearum* management. However, there are serious concerns due to their declining effectiveness and the high selection pressure for fungicide resistance [13,14,15]. Thus, the use of triazole fungicides may potentially be reduced due to a decrease in *Fusarium* sensitivity. Additionally, evidence concerning the endocrine-disrupting potential of triazoles [16,17] has prompted the quest to find alternative safer management strategies. Currently, control strategies such as biopesticides have been gaining interest due to their safer effect on the environment and health. One such approach, “biofumigation”, which was first coined in the 1990s, uses brassica crops such as mustard and radish, which are rich in glucosinolates (GSLs). The method involves growing brassica crops between cash crops, followed by shredding and incorporation into the soil. The tissue disruption allows for the physical contact of GSL and myrosinase enzymes, which are present separately in intact cells [18]. Myrosinase enzymes are located primarily in specialised cells known as myrosin cells, which are dispersed throughout the brassica tissues [19]. Within the myrosin cells, myrosinase enzymes are present in protein containing vacuoles: the myrosin grains [20]. On the other hand, GSLs are distributed throughout the plant organs and are located in translucent vacuoles or moved for long-term storage in sulphur-rich cells called the S-cells [21,22]. Glucosinolates are non-toxic unless they are hydrolysed upon tissue disruption. The interaction of GSL and myrosinases leads to the catalysis of GSL into a range of biologically active substances including toxic isothiocyanates (ITC) [23,24]. These volatile substances are known to have toxic effects on nematodes [25], weed [26], and fungi [27,28].

Although the term biofumigation was introduced in the 1990s, the suppression of soil-borne pathogens by volatile organic compounds from brassica tissues was reported in the 1970/80s. Lewis and Papavizas [29] reported the suppression of *Rhizoctonia solani* using cabbage tissues in the laboratory experiments. Colonisation of buckwheat stem segments in the *R. solani*-infested soil was reduced by 75% when exposed to vapours from decomposing cabbage tissues. Since these initial studies, biofumigation has been investigated for its suppressive effects against fungal pathogens, nematodes, and weeds. In an in vitro study [30], leaf tissues of the *Brassica* species were tested against *Pythium* and *Rhizoctonia* species. Radial growth of *Pythium ultimum* and *Rhizoctonia solani* in Petri dishes were reduced by 100% and 73%, respectively, 48 h after being placed inverted over the neck of a 500 mL jar containing 10 g of macerated leaves of *B. juncea*. In another study [31], where freeze-dried, macerated shoot tissues of brassica cultivars were mixed with 200 g of artificially infested sterile quartz sand (200 *Verticillium dahliae* microsclerotia g^−1^ sand) in sealed flasks, *B. juncea* tissue (0.6 g) proved to be the most inhibitive with up to 80% suppression. Handiseni et al. [32] also successfully demonstrated the suppressive effect of brassica tissues against *R. solani*; in this study, macerated shoot tissue of *B. juncea* (3 g in Petri dish) was found to be the most effective, causing >90% inhibition of mycelial growth.

Whilst biofumigation has attracted significant interest, research on its potential application for reducing the inoculum of *Fusarium* species affecting cereals is scarce. *Fusarium graminearum* produces ascospores (sexual spores) and conidia (asexual spores) and mainly overwinters as mycelium in infected crop debris, which serves as the primary inoculum for head blight disease in cereals [33]. Previously, ITC were tested against mycelial radial growth and conidial germination of *F. graminearum* under in vitro conditions [34]. Among the tested ITC, allyl and methyl ITC were overall more efficient, showing a lower effective dose resulting in 50% inhibition (ED_50_) (35–150 mg L^−1^), suggesting volatiles released from the damaged brassica tissues could have a suppressive effect on *F. graminearum.* Thus, the present study was performed to investigate the potential of brassicas to suppress *F. graminearum*. The aim of this study was to evaluate the effect of the leaf tissue of brassicas on mycelial growth of *F. graminearum* in vitro and to investigate the effect of shredded brassica tissues on the *F. graminearum* inoculum in a closed jar experiment.

## 2. Results

### 2.1. Glucosinolate Content of Brassica Leaf Tissue

The concentrations of the GSLs occurring in the leaf tissue of the brassicas are shown in Table 1. The GSL profile found in the leaves varied both qualitatively and quantitatively among the cultivars. The predominant GSL of *B. juncea* was sinigrin (allyl ITC-precursor) and that of *Raphanus sativus* was glucoraphanin (sulforaphane-precursor). The total GSL concentration of *B. juncea* ‘Brons’ increased with advancing growth stage (24.5–51.9 µmol g^−1^). However, in the other three brassicas tested, the total GSL concentrations in leaf tissue were lower at the early-bud stage compared to stem-extension. Sinigrin comprised 91–94% of the total GSL content of the leaf tissue of *B. juncea* ‘Caliente Rojo’, occurring in the highest concentration (59.5 µmol g^−1^) at the stem-extension stage, while the total GSL concentration ranged from 25.0 to 63.5 µmol g^−1^. The total GSL concentration of *Eruca sativa* ‘Trio ’and *R. sativus* ‘Bokito’ ranged from 12.9 to 17.2 µmol g^−1^ and 8.7 to 39.6 µmol g^−1^, respectively.

### 2.2. Effect of Brassica Leaf Discs on Fusarium graminearum

The effect of *B. juncea* ‘Brons’ on *F. graminearum* varied slightly between the first experiment (with three brassicas; Supplementary Table S1) and the second experiment (with four brassicas). For example, *F. graminearum*, when exposed to two leaf discs collected at the stem-extension stage of *B. juncea* ‘Brons’, showed a 9% reduction in the first experiment in contrast to the 60% reduction in the second experiment. *Raphanus sativus* ‘Bokito’ and *E. sativa* ‘Trio’ showed similar effects in both experiments. Results of the second experiment are presented here.

**Figure 1:**
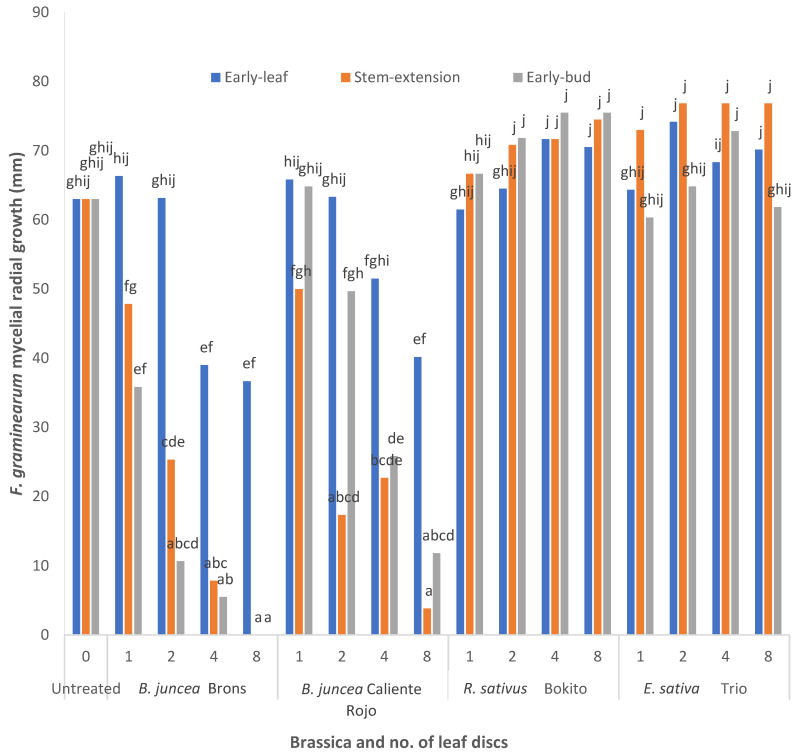
The mycelial colony growth of *Fusarium graminearum* FG2502 in Petri dishes after 5 days untreated and exposed to 1, 2, 4, 8 leaf discs collected at three growth stages of *Brassica juncea* ‘Brons’, *B. juncea* ‘Caliente Rojo’, *Raphanus sativus* ‘Bokito’, and *Eruca sativa* ‘Trio’. Different letters indicate significant differences according to the post hoc Tukey’s test (*p* = 0.05, CV% = 10, SED = 4.276).

**Figure 2:**
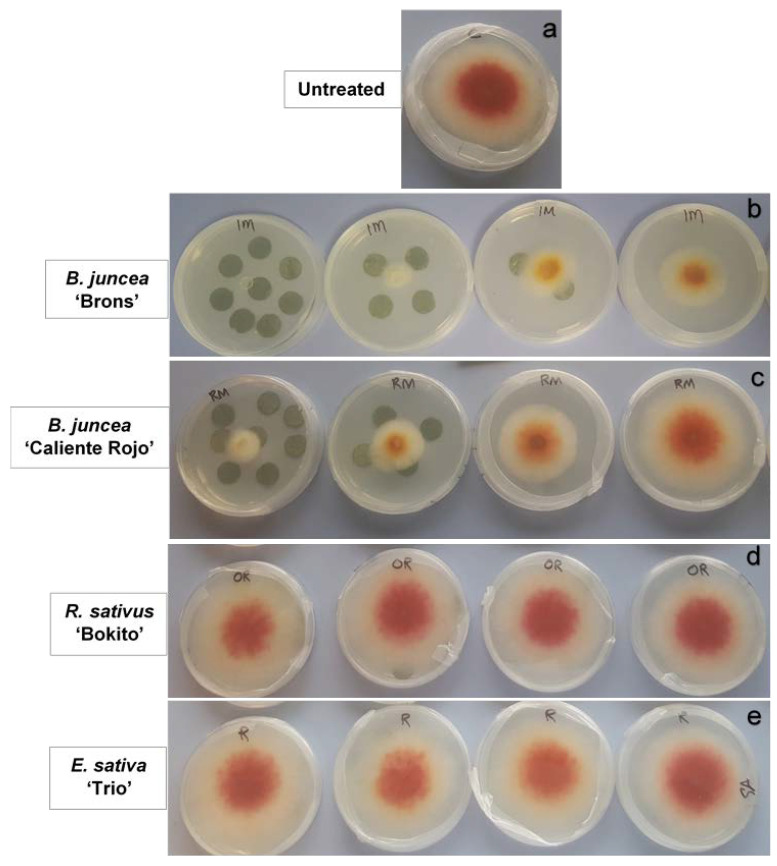
*Fusarium graminearum* FG2502 agar plugs exposed to (**a**) 0 (untreated), 1, 2, 4 and 8 leaf discs (right to left) collected at the early-bud stage of (**b**) *Brassica juncea* ‘Brons’, (**c**) *Brassica juncea* ‘Caliente Rojo’, (**d**) *Raphanus sativus* ‘Bokito’, and (**e**) *Eruca sativa* ‘Trio’, incubated at room temperature (ca. 20 °C) after 5 days.

Different responses of *F. graminearum* were observed according to the brassica species, dosage of leaf discs, and growth stage (Figure 1 and Figure 2). The interaction between the brassica growth stage, brassica species, and number of leaf discs was very highly significant (*p* < 0.001). The fungal growth measured five days after exposure to the *B. juncea* leaf discs indicated a decline in the mycelial growth of *F. graminearum*. At the early-leaf stage experiment, the highest dosage of *B. juncea* ‘Brons’ (eight leaf discs) inhibited the radial growth by 41%. At the stem-extension and early-bud stages, the efficacy of *B. juncea* ‘Brons’ showed 87–90% suppression with four leaf discs, and complete suppression with eight leaf discs. The suppressive effect of all doses at the early-bud stage of *B. juncea* ‘Brons’ was significantly higher (*p* < 0.05) than the control (untreated). *Brassica juncea* ‘Caliente Rojo’ leaf discs collected at the stem-extension stage showed 20% inhibition with the lowest dose (one disc) and 94% inhibition with eight discs. When compared to the untreated control, no significant difference in the radial growth of *F. graminearum* was observed when exposed to leaf discs of *R. sativus* ‘Bokito’ collected at each of the growth stages. In the case of *E. sativa* ‘Trio’ (early-bud stage), the radial growth with one, two, and eight leaf discs was almost similar to that of the untreated, whereas the radial growth with four leaf discs was 15% higher than that of the untreated. At the stem-extension stage, the radial growth with two, four, and eight leaf discs (~20% higher than the untreated) was greater than the radial growth with one leaf disc (15% higher than the untreated). However, the differences in all treatments for *E. sativa* ‘Trio’ were insignificant.

### 2.3. Biofumigation Effect of Brassicas in Closed Jar Experiment

Data on the three brassica species (*B. juncea* ‘Brons’, *R. sativus* ‘Bokito’, *E. sativa* ‘Trio’) was consistent between the first (Supplementary Table S2) and second experiments and the results of the second experiment are presented here.

**Figure 3:**
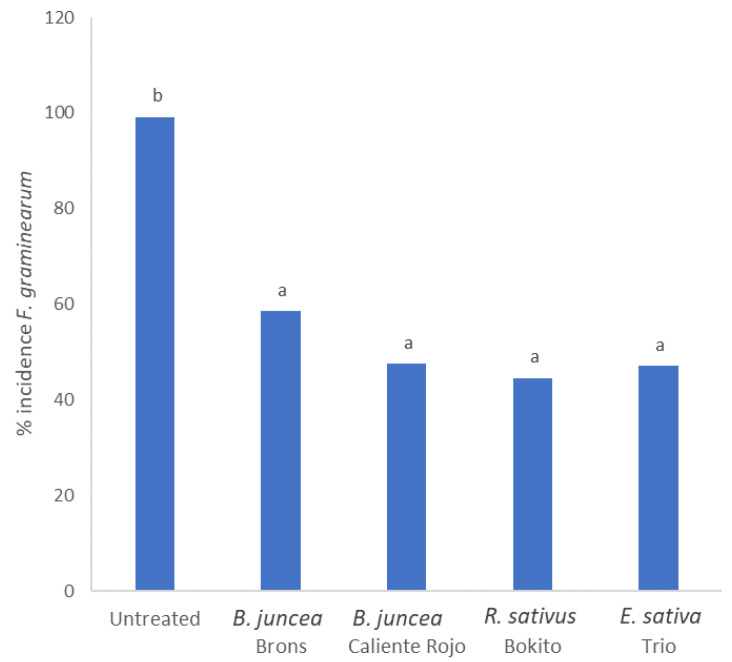
Percentage incidence of *Fusarium graminearum* FG2502 recovered in inoculum incubated for 8 weeks in jars filled with chopped brassica shoots and soil. Different letters indicate significant differences according to the post hoc Tukey’s test (*p* = 0.05, CV% = 60.3, SED = 9.93).

There was no significant interaction between the brassica species, biomass quantity, or inoculum type. The suppressive effect of the chopped shoots of brassicas on the *F. graminearum* inoculum was very highly significant (*p* < 0.001) (Figure 3). On average, inhibition efficiency between 41 and 55% was determined for the brassica treatments tested. The effect of biomass quantity at the two doses (15 g and 65 g) was not significant.

## 3. Discussion

Variation in the efficacy of Brassicaceae plants in inhibiting *F. graminearum* mycelium in the leaf disc assay could be associated with the respective GSL profile. Results highlight that the defrosted leaves of *Brassica juncea* ‘Brons’, collected at the three development stages, caused significant inhibition of the mycelial growth of *F. graminearum*. Meanwhile, the results of the GSL analysis indicate that sinigrin content increases with advanced stages in this cultivar. This could be related to the inhibition of *F. graminearum*, as the level of suppression increased with the advancing growth stage of *B. juncea* ‘Brons’. Similarly, the effect of *B. juncea* ‘Caliente Rojo’ could be related to the sinigrin content detected in the leaf tissue, with the greatest suppression of *F. graminearum* observed with leaves collected at the stem-extension stage. Hence, the effective inhibition of *F. graminearum* mycelium by *B. juncea* leaf discs could be attributed to high levels of sinigrin. Sinigrin is the parent-GSL of allyl ITC and this GSL comprises about 98–99% of the total GSL content of some *Brassica* species such as *B. juncea* and *B. nigra* [35,36]. Allyl ITC has been found to be the predominating compound (>90%) in volatiles released from the macerated leaves of *B. juncea* [30]. Correlations of mycelial inhibition with the release of allyl ITC from brassica leaf tissues have been observed [37]. In a previous study [38], *B. juncea* was found to be the most effective in inhibiting *Sclerotinia sclerotiorum* radial growth by 74–90% when agar plugs were exposed to the fresh macerated tissues of *B. juncea*, *B. campestris*, and *B. napus*. An in vitro assay [39] showed that macerated leaf tissues of *B. juncea* resulted in 73% and 100% inhibition of *F. oxysporum* and *Rhizoctonia solani*, respectively. Conversely, Kirkegaard et al. [40] reported up to 50% suppression of *F. graminearum* mycelial growth by ground freeze-dried tissue of the *B. juncea* shoots (10–500 mg per Petri dish). However, in this study, intact plants were initially frozen at −20 °C before separating into roots and shoots followed by freeze-drying. Hence, the comparatively lower suppression of *F. graminearum* may have resulted from the loss of volatiles by GSL hydrolysis during storage at −20 °C and the processing of samples, in contrast to the present study where leaf discs were immediately flash frozen in liquid nitrogen and stored at −80 °C. Nevertheless, the two studies cannot be directly compared due to the difference in the type of tissue material used.

If we assume a sinigrin content of 48.5 µmol g^−1^ leaf tissue (Table 1) and an ITC release efficiency of 1% [41], eight leaf discs would yield allyl ITC concentrations of 49 mg kg^−1^, suggesting that this concentration is sufficient to completely inhibit *F. graminearum* mycelial growth as shown by the *B. juncea* ‘Brons’ leaves from the early-bud stage. Morra and Kirkegaard [41] recorded 14–26% efficiency of ITC release from *B. juncea* leaf discs frozen at −19 °C prior to incubation with the soil in bottles, whereas a <1% release efficiency was noticed using fresh leaf discs. The higher efficiency was attributed to extreme membrane disruption due to the freezing and thawing of tissues, allowing for greater contact between GSL and myrosinase. Hence, the allyl ITC release efficiency from the frozen leaf discs in the present assay might be higher than 1% due to a greater GSL/ITC conversion. Previous in vitro work has shown the suppressive effect of allyl ITC on *F. graminearum* at ED_50_ concentrations of 62–135 mg L^−1^ [34]. Studies on *Alternaria* spp. suggest that ITC promotes the production of reactive oxygen species and disrupts mitochondrial function [42] and the plasma membrane [43,44] in fungal cells. In our previous study [34], the sensitivity of five strains of *F. graminearum* (FG2556, FG2498, FG2560, FG2502, FG2481—from UK wheat isolated in 2016) to ITC associated with these brassicas was found to be broadly similar. Therefore, in the present study, one strain, FG2502, was assessed.

In the leaf disc assay, *F. graminearum* mycelial plugs, which were completely inhibited by *B. juncea* ‘Brons’ treatments, showed no subsequent growth when transferred to fresh potato dextrose agar (PDA) media, indicating that the effect was fungicidal rather than fungistatic (data not shown). This is consistent with Charron and Sams [30], who reported a fungicidal effect of *B. juncea* macerated leaves on the radial growth of *Pythium ultimum*. The radial growth of *P. ultimum* exposed to macerated leaves was completely inhibited after 48 h, and the *P. ultimum* plugs, when transferred to fresh PDA, did not grow. The lowest dose (one leaf disc) of *B. juncea* showed a slight stimulation in colony growth, although this was not significantly different to the control. *Raphanus sativus* ‘Bokito’ and *E. sativa* ‘Trio’ also appeared to insignificantly stimulate colony growth. Such stimulation is consistent with a previous report by Kirkegaard et al. [40] where the colony growth of *Bipolaris sorokiniana* was stimulated when exposed to lower quantities of *B. juncea* and *B. napus* tissues. A slightly higher inhibition in the *B. juncea* ‘Brons’ treatment was seen in the second experiment in comparison to the first leaf disc assay, as above-mentioned. This could be due to plants grown at different times of the year (experiment 1: October–January; experiment 2: September–November). Slightly longer daylight hours and higher temperatures during the time period of the second experiment may have resulted in higher GSL concentrations, as these factors are known to increase the production of GSL in brassica tissues [45].

The two cultivars of *B. juncea* were effective in both the leaf disc and closed jar experiments, however, *R. sativus* and *E. sativa*, despite being ineffective in the leaf disc assay, showed significant suppression in the closed jar experiment. In addition to ITC, other less toxic compounds such as nitriles and thiocyanates are also produced as a result of GSL hydrolysis, and these compounds are known to have biocidal properties [46,47]. Moreover, other toxic compounds such as dimethyl disulphide and carbon disulphide are released during the decomposition of plant material, which may also contribute to the biofumigation effect [48,49]. These factors may have contributed to the suppressive activity seen by *R. sativus* and *E. sativa* in the present closed jar study. 

In the closed jar experiment, the two quantities of the chopped tissue added, 65 g and 15 g, were calculated as equivalent to 50 t fresh wt. ha^−1^ and 12 t fresh wt. ha^−1^, respectively. The rationale behind these rates was to mimic the biofumigation potential of brassica plants with an achievable high biomass (50 t fresh wt. ha^−1^) in the field [50] and a relatively low biomass (25% of a high yield). Previously, Handiseni et al. [32] exposed *R. solani* mycelial plugs to *B. juncea*-amended soil in sealed bags. A dose dependent response was reported with inhibition ranging from <5% inhibition at a 0.4% (wt/wt) incorporation rate to approximately 50% inhibition at the 3.2% (wt/wt) incorporation rate. This was in contrast to findings from the closed jar experiment where the dose effect was not significant. As the present experiment was carried out in airtight jars, volatiles released from even the lower dose (15 g) were sufficient to effectively suppress *F. graminearum*. However, in agreement with the present findings, Mayton et al. [37] reported a >50% radial growth inhibition of *F. sambucinum* in Petri dishes inverted onto jars containing the macerated leaf tissue of *B. nigra* and *B. juncea*, which was not affected by the quantity of tissue (10–40 g). 

Using a cut-and-carry approach, brassica mulch was applied to *F. graminearum* infected wheat plots in field experiments performed over two years [51]. Fusarium head blight incidence was significantly reduced by 58% in the first year by *Sinapis alba* and 18% by *B. juncea* in the second year. The two types of mulch also reduced deoxynivalenol content in the wheat grain by 40–50%. Crop debris, particularly that of maize, is a primary source of inoculum for Fusarium head blight in wheat [52,53]. Crop debris is present in all shapes and sizes, and to reduce the variability within an experimental system, it is beneficial to have an artificial crop debris model system. Two types of substrates were used in the present study, chaff represented natural crop residue and blind oat spikes represented an artificial crop debris that is uniform in size and nutritional status, and were uniformly infected with *F. graminearum*. The volatiles from the chopped brassica tissue appeared to have inhibited *F. graminearum* inoculum, irrespective of the inoculum type. This suggests that biofumigation could prove to be effective in reducing the *F. graminearum* inoculum present in a variety of crop residues such as chaff, seed, and straw under field conditions. However, it would be useful to identify whether biofumigation is equally effective against inoculum of various sizes, as *F. graminearum* within larger pieces of inoculum may be protected from contact with the inhibitory compounds released during the process.

*Brassica juncea* ‘Caliente Rojo’, which is a fairly new cultivar, was not available at the time period of the first experiments. The suppressive effect of *B. juncea* ‘Caliente Rojo’ seen in the present study suggests that this cultivar could be a promising biofumigant for managing *F. graminearum* in the field. The cultivar could be sown following the harvest of wheat in summer, and incorporated in autumn followed by subsequent cereal production. Glucosinolate analysis of this cultivar showed that concentrations of the dominant GSL, sinigrin, were highest at the stem-extension stage compared to the other two stages. A balance between the biomass and GSL concentration could be achieved by identifying an appropriate growth stage between stem-extension and flowering to maximize the GSL content for incorporation. The biofumigant, when chopped and incorporated, is then likely to produce ITC at sufficiently effective concentrations. Glucosinolate concentrations recorded in the present study could be achievable in field situations. Total GSL concentrations of up to 50, 69, and 61 µmol g^−1^ biomass have been detected in *B. juncea*, *R. sativus*, and *E. sativa*, respectively under field conditions [54]. Biofumigation, if applied in a cereal rotation, may therefore be useful in the reduction of *F. graminearum* inoculum in the field, thus suppressing infection in the subsequent cereal crop. However, it should be considered that the present experiments were carried out under controlled conditions. In the field, various factors such as soil pH, temperature, moisture, and organic content as well as the biofumigant crop establishment and growth should be considered, as these factors can affect the outcome of biofumigation. Moreover, weather conditions such as temperature, daylight hours, and rainfall also influence the efficacy of biofumigation.

## 4. Materials and Methods

### 4.1. Brassicas and Fungal Culture

Seeds of *B. juncea* ‘Brons’ (Indian mustard), *R. sativus* ‘Bokito’ (oilseed radish), and *E. sativa* ‘Trio’ (rocket) were supplied by RAGT Seeds (Essex, UK) and *B. juncea* ‘Caliente Rojo’ (Indian mustard) by Tozer Seeds (Surrey, UK). The four cultivars were grown in a glasshouse in 10 cm pots with mean temperatures of 12 °C (night) and 17 °C (day). For first experiment, plants were grown between October 2019–January 2020 and for second experiment, plants were grown between September–November 2020. *Fusarium graminearum* strain FG2502 from UK wheat isolated in 2016 was provided by Fera Science Ltd. (York, UK). The strain was confirmed as *F. graminearum* by species-specific PCR using a previously published assay [55].

### 4.2. Leaf Disc Assay

Leaves of three cultivars (*B. juncea* ‘Brons’, *R. sativus* ‘Bokito’, *E. sativa* ‘Trio’) were sampled at the early-leaf stage (4–5 true leaves unfolded). Using a 15 mm (diameter) cork borer, 45 discs were cut from the leaves of each cultivar. The leaf discs, separated by Miracloth (EMD Millipore Corp., Billerica, MA, USA) in stacks of fives and held together by a paper clip, were immediately flash frozen in liquid nitrogen and stored at −80 °C until required for the assay. Agar plugs with a 7 mm diameter were cut from the outer margin of actively growing *F. graminearum* (FG2502) on PDA (Merck, KGaA, Darmstadt, Germany) media. These were transferred to the centre of 9 cm Petri dishes containing PDA with the mycelium facedown. The frozen leaf discs were transferred in dry ice from the freezer to the work-station to prevent the discs from defrosting prematurely. Leaf discs at doses of one, two, four, and eight discs were placed with forceps onto the upturned lids of the Petri dishes while the inverted bottom containing the fungal plug was held aside. The plates in the inverted position were immediately sealed with parafilm. The control plates did not contain leaf discs. Three replicates were used per treatment. The plates were incubated at room temperature (ca. 20 °C) and radial colony growth was measured after 5–6 days, just before the untreated mycelia had reached the edge of the plates. Experiments to investigate the effect of brassica leaves at the stem-extension and early-bud stages were performed as above.

The assay was repeated independently using *B. juncea* ‘Caliente Rojo’, in addition to the other brassica species tested in the first experiment.

### 4.3. Closed Jar Experiment

#### 4.3.1. Preparation of Inoculum Bags

Conidial suspensions were prepared following a previous method [56] with some modifications. *Fusarium graminearum* (FG2502) was sub-cultured on PDA using 20 plates and incubated at room temperature (ca. 20 °C). After 14 days, conidia were harvested by the addition of 20–25 mL sterile distilled water to each plate and conidia were dislodged using a sterile spreader. The suspension was filtered through Miracloth to remove the mycelium. The filtrate was centrifuged at 3000× *g* for 10 min. The supernatant was removed and conidia re-suspended in 15 mL sterile distilled water and centrifuged at 3000× *g* for 10 min. Conidia were washed twice and re-suspended in 15 mL sterile distilled water. Conidia were counted using an Improved Neubauer counting chamber (Weber 99, Scientific International, Teddington, UK) and the concentration adjusted to 3 × 10^4^ spores mL^−^^1^. 

**Figure 4:**
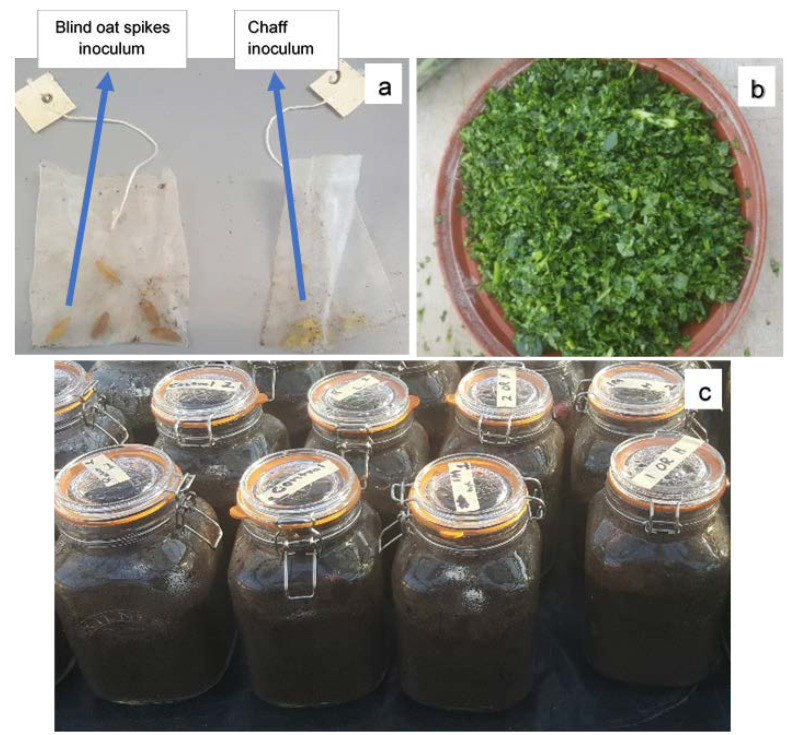
The experimental setup of the closed jar biofumigation experiment. (**a**) Blind oat spikes inoculated with *Fusarium graminearum* and chaff from the *F. graminearum*-inoculated wheat in nylon sachets. (**b**) Brassica shoots chopped in food processor. (**c**) Sealed jars containing the two types of inoculum sachets buried in loam-based compost incorporated with chopped brassica tissue.

A kilogram of oat screenings (Morning Foods, Crewe, UK), which were predominantly blind oat spikes, were soaked in distilled water for 12 h before autoclaving for 15 min in a 78 cm × 40 cm autoclavable bag [VWR (129–0581), UK]. The oats were re-autoclaved for 15 min after 24 h and allowed to cool. A total of 100 mL (3 ×10^6^ spores) of the above prepared spore suspension was added to the bag, mixed well, and incubated for 10–12 days in the dark at ca. 18 °C. Five pieces of inoculum (blind oat spikes) were added to perforated nylon sachets (7 cm × 5.5 cm) and sealed with a plastic bag sealing machine (Impulse heat sealer, 200 mm). Additionally, wheat chaff was randomly collected post-harvest from a *F. graminearum*-inoculated wheat experiment at the research facilities of Harper Adams University, Newport, Shropshire, UK. Five pieces of chaff were added to nylon bags and sealed as described above. Both types of inoculum (Figure 4a) were confirmed for *F. graminearum* infection on PDA prior to use in the experiment.

#### 4.3.2. Evaluating Effect of Biofumigants on Inoculum

Two litre glass jars with rubber seal clips (22 cm × 12 cm × 12 cm) were filled with John Innes No. 2 loam-based compost [composition: loam, peat, coarse sand, hoof and horn meal, superphosphate, potassium sulphate, calcium carbonate; pH: 5.5–6 (provided by supplier); 46% moisture] to a depth of 10 cm. At the early-bud stage, shoots of *B. juncea* ‘Brons’, *R. sativus* ‘Bokito’, and *E. sativa* ‘Trio’ were harvested and chopped in a food processer for 15 s. Chopped tissue (Figure 4b) at two quantities, 65 g and 15 g, were added to soil-filled jars and mixed well. Jars without brassica amendments served as the controls. One inoculum bag (blind oat spikes) was buried in each jar and the lid closed hermetically. Five replicates were used per treatment and the jars were incubated in a glasshouse with mean temperatures of 12 °C (night) and 17 °C (day) (Figure 4c). After eight weeks, the bags were extracted, and the inoculum substrates were tested for *F. graminearum* growth on Petri dishes containing modified Czapek Dox iprodione agar media [57]. Briefly, the selective media were prepared by adding dichloran solution (0.2% in ethanol), chloramphenicol solution (5% in ethanol), and trace metal solution (1% ZnSO_4_∙7H_2_O + 0.5% CuSO_4_∙5H_2_O) each 1 mL L^−1^ of Czapek Dox agar (Sigma-Aldrich, Switzerland) media. Once autoclaved and cooled down to 55 °C, 10 mL of chlortetracycline solution (0.5%) and 1 mL of Bumper^®^ suspension (0.3%) containing 750 µg propiconazole were added per L of media before pouring into plates. The five inoculum pieces were removed from each bag, placed on a media plate, and incubated at room temperature (ca. 20 °C) for 12–15 days. *Fusarium graminearum* incidence in the inoculum pieces was recorded as the presence of *F. graminearum* colony growth based on the characteristic reddish pigmentation. For confirmation, the assumed *F. graminearum* colonies were sub-cultured on PDA and incubated at room temperature (ca. 20 °C) for 14 days. The conidia were harvested as described above and identified according to the morphological characteristics [58].

The experiment was independently repeated using *B. juncea* ‘Caliente Rojo’, in addition to the other brassicas tested in the first experiment. Additionally, a bag of the chaff inoculum (five pieces per bag) was also buried in each jar.

### 4.4. Glucosinolate Analysis

Approximately 10 g of leaf tissue of the brassica plants was collected at the three developmental stages (early-leaf, stem-extension, early-bud), flash frozen in liquid nitrogen, and stored at −80 °C until freeze dried. Freeze-dried (GVD6/13 MKI freeze dryer; GIROVAC Ltd., North Walsham, UK) samples were milled (IKA^®^M 20 Universal Mill, Staufen, Germany) and stored at −18 °C before sending to the NIAB Labtest, Cambridge, UK, where the samples were analysed following the ISO 9167 “Rapeseed and rapeseed meals—Determination of glucosinolates content—Method using HPLC” [59].

### 4.5. Statistical Analysis

Data were subjected to general analysis of variance using Genstat^®^ (20th edition) statistical software. The closed jar experiments were arranged in the glasshouse in a completely randomised design. In the leaf disc assay, there were three factors—brassica species, brassica growth stage, number of leaf discs. In the closed jar experiment, the three factors were brassica species, biomass quantity, and inoculum type. Where necessary, data were angular transformed to improve the normality of the residuals. Significant differences between treatments were determined using the post hoc Tukey test (*p* = 0.05).

## Figures and Tables

**Table 1:** The type and concentration of glucosinolates in freeze-dried leaf tissue of brassica plants used in the leaf disc assay to investigate the effect of volatiles released from defrosted leaf discs of brassicas on the mycelial growth of *Fusarium graminearum* in vitro.

Glucosinolate	*Brassica juncea* Brons			*Brassica juncea* Caliente Rojo			*Raphanus sativus* Bokito			*Eruca sativa* Trio		
	I ^a^	II ^b^	III ^c^	I	II	III	I	II	III	I	II	III
Glucoberin	2.47 (0.6) ^d^	3.15 (0.49)	2.01 (0.41)	1.29 (0.2)	1.81 (0.3)	1.33 (0.37)	2.28 (0.4)	1.16 (0.18)	2.15 (0.6)	2.54 (0.6)	2.55 (0.33)	2.37 (0.75)
Progoitrin	0.38 (0.1)	0.34 (0.02)	0.30 (0.10)	0.25 (0.04)	0.23 (0.11)	0.26 (0.09)	0.35 (0.08)	0.37 (0.12)	0.46 (0.08)	0.30 (0.1)	0.00	0.00
Sinigrin	21.44 (1.7)	24.02 (2.94)	48.52 (3.03)	22.72 (1.88)	59.54 (2.32)	50.9 (3.8)	-	-	-	-	-	-
Gluconapin	0.00	0.00	0.35 (0.09)	0.27 (0.07)	0.35 (0.03)	0.25 (0.09)	-	-	-	-	-	-
Glucobrassicin	0.14 (0.04)	0.03 (0.01)	0.11 (0.02)	0.11 (0.02)	0.14 (0.02)	0.23 (0.12)	1.62 (0.26)	3.87 (1.9)	2.62 (0.6)	0.22 (0.03)	0.54 (0.08)	0.58 (0.08)
Gluconasturtiin	0.11 (0.01)	0.39 (0.05)	0.58 (0.08)	0.24 (0.08)	0.97 (0.19)	0.76 (0.12)	0.32 (0.13)	1.13 (0.2)	0.96 (0.2)	0.77 (0.12)	0.42 (0.15)	0.24 (0.10)
Neoglucobrassicin	0.00	0.00	0.00	0.05 (0.01)	0.04 (0.01)	0.01 (0.007)	0.00	0.00	0.00	0.00	0.06 (0.02)	0.00
Glucoraphanin	-	-	-	-	-	-	2.90 (0.5)	23.56 (3.2)	7.29 (2.1)	2.42 (0.51)	2.84 (0.64)	2.75 (0.6)
Glucoraphenin	-	-	-	-	-	-	0.18 (0.05)	1.34 (0.4)	0.44 (0.07)	-	-	-
4 hydroxy glucobrassicin	-	-	-	0.05 (0.01)	0.37 (0.1)	0.4 (0.15)	0.00	0.04 (0.01)	0.03 (0.01)	-	-	-
Glucoraphasatin	-	-	-	-	-	-	1.02 (0.03)	8.10 (1.1)	11.09 (3.1)	-	-	-
Glucoalyssin	-	-	-	-	-	-	-	-	-	0.86 (0.18)	0.51 (0.2)	0.45 (0.14)
Glucoerucin	-	-	-	-	-	-	-	-	-	0.57 (0.14)	1.20 (0.01)	1.50 (0.28)
4-mercaptobutyl	-	-	-	-	-	-	-	-	-	3.58 (0.72)	5.70 (0.44)	3.21 (0.58)
unknown	-	-	-	-	-	-	-	-	-	0.61 (0.2)	0.83 (0.23)	0.69 (0.19)
unknown	-	-	-	-	-	-	-	-	-	1.04 (0.12)	2.59 (0.66)	2.83 (0.74)
Total glucosinolates	24.54 (2.26)	27.94 (4.18)	51.87 (3.34)	24.98 (4.59)	63.45 (3.46)	54.15 (2.94)	8.67 (2.33)	39.56 (1.25)	25.05 (3.69)	12.90 (1.20)	17.23 (2.47)	14.63 (2.57)

^a^ I = early-leaf growth stage (4 to 5 true leaves unfolded); ^b^ II = stem-extension stage; ^c^ III = early-bud stage; ^d^ GSL conc. µmol g^−1^ freeze-dried leaf tissue, mean (SE) values from two replicates.

## Data Availability

All data generated or analysed during this study are included in the published article.

## Supplementary Materials

The following are available online at https://www.mdpi.com/article/10.3390/antibiotics11091249/s1, Table S1. Mycelial colony growth (mm) of *Fusarium graminearum* FG2502 in Petri dishes after 5 days untreated and exposed to 1, 2, 4, 8 leaf discs collected at three growth stages of *Brassica juncea* ‘Brons’, *Raphanus sativus* ‘Bokito’, and *Eruca sativa* ‘Trio’ in the leaf disc assay (first experiment). Table S2: Percentage incidence of *Fusarium graminearum* FG2502 recovered in inoculum (mean values) incubated for 8 weeks in jars filled with chopped brassica shoots and soil in the closed jar assay (first experiment).
