# Supplementary material for: Antifungal Effect of Brassica Tissues on the Mycotoxigenic Cereal Pathogen Fusarium graminearum
Source: Antibiotics (Basel). 2022 Sep 15;11(9):1249. doi: 10.3390/antibiotics11091249 (PMC9495792; doi:10.3390/antibiotics11091249)
Supplement: Supplementary file 1 [file antibiotics-11-01249-s001.zip › antibiotics-1877100-supplementary.pdf]

**Supplementary Table S1.** Mycelial colony growth (mm) of *Fusarium graminearum* FG2502 in Petri dishes after 5 days untreated and exposed to 1, 2, 4, 8 leaf discs collected at three growth stages of *Brassica juncea* 'Brons', *Raphanus sativus* 'Bokito' and *Eruca sativa* 'Trio' in leaf disc assay (first experiment)

| Treatments | No. of leaf discs | Early-leaf stage | Stem-extension stage | Early-bud stage |
|---|---|---|---|---|
| | | Mean mycelial colony growth (mm) of *Fusarium graminearum* | | |
| Untreated | 0 | 75 | 75 | 75 |
| *Brassica juncea* 'Brons' | 1 | 77 | 76 | 37 |
| | 2 | 73 | 68 | 33 |
| | 4 | 76 | 51 | 19 |
| | 8 | 48 | 8 | 0 |
| *Raphanus sativus* 'Bokito' | 1 | 74 | 75 | 75 |
| | 2 | 76 | 76 | 75 |
| | 4 | 77 | 76 | 76 |
| | 8 | 77 | 75 | 76 |
| *Eruca sativa* 'Trio' | 1 | 74 | 75 | 74 |
| | 2 | 77 | 75 | 75 |
| | 4 | 77 | 76 | 76 |
| | 8 | 76 | 76 | 76 |

**Supplementary Table S2.** Percentage incidence of *Fusarium graminearum* FG2502 recovered in inoculum (mean values) incubated for 8 weeks in jars filled with chopped brassica shoots and soil in closed jar assay (first experiment)

| **Treatments** | **% incidence *Fusarium graminearum* recovered in inoculum** |
|---|---|
| Untreated | 100 |
| *Brassica juncea* 'Brons' | 56 |
| *Raphanus sativus* ‘Bokito’ | 52 |
| *Eruca sativa* ‘Trio’ | 33 |
